# Using Natural Language Processing to Explore Social Media Opinions on Food Security: Sentiment Analysis and Topic Modeling Study

**DOI:** 10.2196/47826

**Published:** 2024-03-21

**Authors:** Annika Molenaar, Dickson Lukose, Linda Brennan, Eva L Jenkins, Tracy A McCaffrey

**Affiliations:** 1 Department of Nutrition, Dietetics and Food Monash University Notting Hill Australia; 2 Tabcorp Holdings Ltd Melbourne Australia; 3 School of Media and Communication RMIT University Melbourne Australia

**Keywords:** food security, food insecurity, public health, sentiment analysis, topic modeling, natural language processing, infodemiology

## Abstract

**Background:**

Social media has the potential to be of great value in understanding patterns in public health using large-scale analysis approaches (eg, data science and natural language processing [NLP]), 2 of which have been used in public health: sentiment analysis and topic modeling; however, their use in the area of food security and public health nutrition is limited.

**Objective:**

This study aims to explore the potential use of NLP tools to gather insights from real-world social media data on the public health issue of food security.

**Methods:**

A search strategy for obtaining tweets was developed using food security terms. Tweets were collected using the Twitter application programming interface from January 1, 2019, to December 31, 2021, filtered for Australia-based users only. Sentiment analysis of the tweets was performed using the Valence Aware Dictionary and Sentiment Reasoner. Topic modeling exploring the content of tweets was conducted using latent Dirichlet allocation with BigML (BigML, Inc). Sentiment, topic, and engagement (the sum of likes, retweets, quotations, and replies) were compared across years.

**Results:**

In total, 38,070 tweets were collected from 14,880 Twitter users. Overall, the sentiment when discussing food security was positive, although this varied across the 3 years. Positive sentiment remained higher during the COVID-19 lockdown periods in Australia. The topic model contained 10 topics (in order from highest to lowest probability in the data set): “Global production,” “Food insecurity and health,” “Use of food banks,” “Giving to food banks,” “Family poverty,” “Food relief provision,” “Global food insecurity,” “Climate change,” “Australian food insecurity,” and “Human rights.” The topic “Giving to food banks,” which focused on support and donation, had the highest proportion of positive sentiment, and “Global food insecurity,” which covered food insecurity prevalence worldwide, had the highest proportion of negative sentiment. When compared with news, there were some events, such as COVID-19 support payment introduction and bushfires across Australia, that were associated with high periods of positive or negative sentiment. Topics related to food insecurity prevalence, poverty, and food relief in Australia were not consistently more prominent during the COVID-19 pandemic than before the pandemic. Negative tweets received substantially higher engagement across 2019 and 2020. There was no clear relationship between topics that were more likely to be positive or negative and have higher or lower engagement, indicating that the identified topics are discrete issues.

**Conclusions:**

In this study, we demonstrated the potential use of sentiment analysis and topic modeling to explore evolution in conversations on food security using social media data. Future use of NLP in food security requires the context of and interpretation by public health experts and the use of broader data sets, with the potential to track dimensions or events related to food security to inform evidence-based decision-making in this area.

## Introduction

### Background

Social media has become ubiquitous for people creating and sharing information, news, and experiences in real time, including communicating about issues such as health and nutrition. This engagement on social media creates a vast amount of information that is continually being updated—all day, every day. Deciphering large volumes of information, such as that from social media, can help inform future public health practices based on the current state of affairs, track disease outbreaks, reduce health misinformation, encourage social mobilization by understanding what is important to the public, and highlight future directions in health care [1]. This study used food security as an example of a complex and prevalent public health issue.

Food security can be defined as the availability of and physical, social, and financial access to sufficient, safe, culturally appropriate, and nutritionally adequate food [2,3]. Data science and machine learning techniques (Multimedia Appendix 1 [4-15]) present opportunities to analyze and interpret large-scale public health data to gain an understanding of what is being discussed about food security, in what way, and by whom. Machine learning can classify real-world data such as discussions on social media about food security through statistical models and algorithms built from the analyzed data [16]. One area of data science and machine learning of particular interest in social media analysis is natural language processing (NLP). NLP techniques are able to learn and understand human language [4] and, therefore, can explore the opinions and real-life experiences of social media users through their web-based conversations related to public health issues such as food security [17].

At the public health level, the use of electronic media such as social media for information gathering to understand and inform public health is known as infodemiology [5,18]. One of the goals of infodemiology is to collect and evaluate information on the web (often using data science techniques) that is related to public health, including public communication patterns and behaviors related to a public health issue [5]. Alongside infodemiology is infoveillance, which refers to the use of web-based information for surveillance purposes such as tracking public health events [5]. Infodemiology and infoveillance were key techniques used during the COVID-19 pandemic and vaccination rollout [19]. For example, infodemiology and infoveillance were used to classify and explore misinformation about COVID-19 [20], explore public discourse on COVID-19 and vaccinations [21,22], and track COVID-19 cases and deaths [23]. COVID-19 also highlighted the issue of misinformation and the emergence of an infodemic (Multimedia Appendix 1), with users having access to vast amounts of information, misinformation, and disinformation during the pandemic [24]. Public health professionals, alongside data scientists and behavior change experts, play a role in understanding the theories regarding misinformation and the strategies that can be used to monitor and mitigate the spread of health misinformation, particularly using digital technologies and social media [25].

A commonly used NLP technique to interpret social media data in infodemiology is sentiment analysis (Multimedia Appendix 1), which enables understanding of the discourse on a topic [26]. Sentiment analysis—sometimes referred to as “emotion analysis,” “subjectivity analysis,” or “opinion mining”—analyzes the opinions, sentiments, attitudes, and emotions embodied within written forms of natural language (eg, social media data) [6]. One review found that sentiment analysis was used in 12 studies in the area of health care to analyze Twitter data with different sentiment analysis tools ranging from open-source publicly available tools to tools produced specifically for the study [27]. Sentiment analysis was also used in 86 studies in the areas of health and well-being. These studies used data from social networking sites and web-based retail platforms and covered a wide range of topics, for example, health conditions, health treatments, mental health, and quality of life [28]. Previous research on social media related to nutrition has largely focused on engagement (eg, likes, shares, and comments) on a small scale (between 9 social media profile pages and 736 social media posts) using manual analysis by topic experts [29-31] and has less frequently explored the breadth of the public’s opinions and emotions expressed in social media posts. More recently, sentiment analysis tools, along with additional data science techniques such as topic modeling and social network analysis, were used to explore many nutrition-related topics on social media across 37 studies [32]. Using sentiment analysis alongside other NLP techniques enables researchers to gain a more in-depth understanding of large data sets such as those created in social media, thus providing further insights into potential implications for public health.

Topic modeling is an NLP process that is able to sort textual data (eg, social media data) into different themes or categories of topics using probabilistic algorithms [7,33]. One goal of infodemiology is to explore co-occurrences of different concepts of real-world social media data [5]; this is achieved through topic modeling, which groups text-based data into themes through co-occurrences of words and concepts. Topic modeling can use large data sets to explore relationships between themes of conversation and changes over time through topic evolution [33]. Topic modeling can also be used to track the evolution of discussions across time, taking snapshots of data at different time points and comparing the sentiment, emotion, or topic analyses at each time point. Topic modeling has been used to characterize specific areas of health that social media users commonly discuss on social media platforms [34,35], and recently, topic modeling has been the focus of a great deal of research exploring the discourse on the COVID-19 pandemic through news and social media sources [36-38]. As a social media analysis tool, topic modeling has the potential to categorize and explore real-time opinions, beliefs, and attitudes in a real-world public health context.

Thus far, infodemiology and infoveillance studies have primarily focused on disease states, outbreaks and epidemics, health care, drugs, smoking, alcohol, and mental health, with less focus on nutrition-related public health issues [26]. Creating methodological processes for gathering information to inform practice or policy has been an urgent focus in research on communicable diseases such as COVID-19. However, the focus on such NLP processes does not exist in areas of complex, multifaceted public health issues such as food security despite its importance to overall health and well-being and the pervasiveness across different population groups.

The concept of food security is underpinned by different dimensions related to access to food and the stability of these dimensions, a population or individual’s food access and availability, the ability to use the nutrition from the food [3], agency to influence the food system, and the sustainability of the food from both a social and ecological perspective [39]. The term “food security” refers to when the dimensions have been achieved, and the term “food insecurity” refers to when all these dimensions have not been achieved. The prevalence of food insecurity and subsequent malnutrition worldwide has been increasing [40], with most undernourished people being from low- and middle-income countries in Asia, where 381 million people experience food insecurity, and Africa, where >250 million people experience food insecurity [41]. In high-income countries, the health effects of food insecurity are varied; in adults, they include the development of chronic diseases and obesity [42,43], mental illness, and social isolation [44,45], and in children, they include poor physical and academic development and behavioral issues [46]. Owing to its prominence and the effects it has on nutrition and health, food security is the focus of one of the United Nations Sustainable Development Goals, that is, the goal of ending hunger, achieving food security, improving nutrition, and promoting more sustainable agriculture by 2030 [40].

As with most public health issues, high-income countries are not immune to the effects of food insecurity; for example, it is estimated that 4% to 13% of Australians are affected by food insecurity [47]. Unfortunately, in Australia, there is a lack of a national coordinated response to address food insecurity, with most interventions being at the level of the state or local council area [48]. In addition, the current focus for addressing food insecurity relies on food relief and food banks and, therefore, does not address or seek to further understand the systemic causes of food insecurity [49,50] or the changing issues related to food access arising from events such as the COVID-19 pandemic [51]. This makes information and insight gathering potentially difficult, with widespread and differing reporting on the prevalence of as well as response to the issue of food security. Given that the response to food insecurity in Australia is potentially falling short owing to the overall prevalence and lack of coordinated action, there is a need for new strategies. To gather real-world insights to help support and inform such strategies and decision-making in the area, new data sources, including those of large scale and with real-time updates, should be explored. This has been done in previous research that used artificial intelligence and NLP to use data to predict crop yield and, therefore, assist with cropland mapping to enhance food production and improve access, which is one dimension of food security [52,53]. Other research has highlighted how machine learning can assist in exploring complex socioeconomic parameters related to food security and the interactions among key agents such as climate change, food price dynamics, social networks, and food markets [53].

### Objectives

Given that the issue of food security is broad, complex, and multifaceted, this study explored all dimensions of food security with a focus on the Australian context. To be able to capture real-world opinions through social media related to a complex public health issue such as food security, it is crucial to have a deep understanding of the necessary methodological processes. As previous research has not used both the NLP techniques of sentiment and topic analysis on social media data related to food security, the research objective was to gather insights into the potential of these methods in this area. Therefore, the aim of this study was to explore the value of using NLP tools to gather insight from real-world social media data on food security. This study constitutes one of the steps toward using real-world data sources to build infoveillance in public health areas such as food security, with the ultimate aim of enabling evidence-based decision-making for public health professionals. Infoveillance-informed decision-making regarding food security has the potential to create interventions that can keep up with real-time changes in the area and are informed by a broad range of stakeholders, including people experiencing food insecurity.

## Methods

### Search Term Development

Twitter was chosen as the data collection platform in January 2021 because of its text-based nature, which is suitable for NLP, and the large amounts of publicly available data that can be accessed through the Twitter application programming interface (API; Multimedia Appendix 1). It is acknowledged that Twitter represents only a subset of the population, and therefore, the analysis will not be representative of the broader population of social media users. Nonetheless, the procedures outlined in this paper can be applied to other large data sets from social media.

An iterative process of search term development was used to identify a Twitter search term strategy that retrieved tweets relevant to the topic of food security. Publicly available social media posts related to food security were mined from Twitter using the Twitter API. The initial search terms included words related to food security, food banks, and food relief and relevant hashtags such as #zerohunger, #feedthehungry, and #foodforall, which were determined through manual identification of key tweets in the area of food security. Tweets from users indicating that they were from Australia (ie, location in their Twitter biography) were collected for a week using each iteration of search terms. This data set with a week of tweets containing the search term list was manually assessed for relevance to the topic of food security. The search terms were subsequently refined based on search terms that produced irrelevant tweets, and additional terms were included to gather more relevant tweets that were not captured using other search terms. Search terms for data collection went through 5 iterations, with manual relevance coding of between 300 and 535 tweets posted from the previous week over 5 different weeks, to ascertain terms that were included in relevant tweets. The following search terms were in the final list included in the API call for data collection: “food security,” “food insecurity,” “foodbank,” “food bank,” “food relief,” “food insecure,” “food secure,” “food shelter,” #foodsecurity, #foodinsecurity, #foodinsecure, #foodsecure, #foodequity, #zerohunger, #endhunger, #foodforall, #feedthehungry, and #foodbank.

### Data Collection

**Figure 1:**
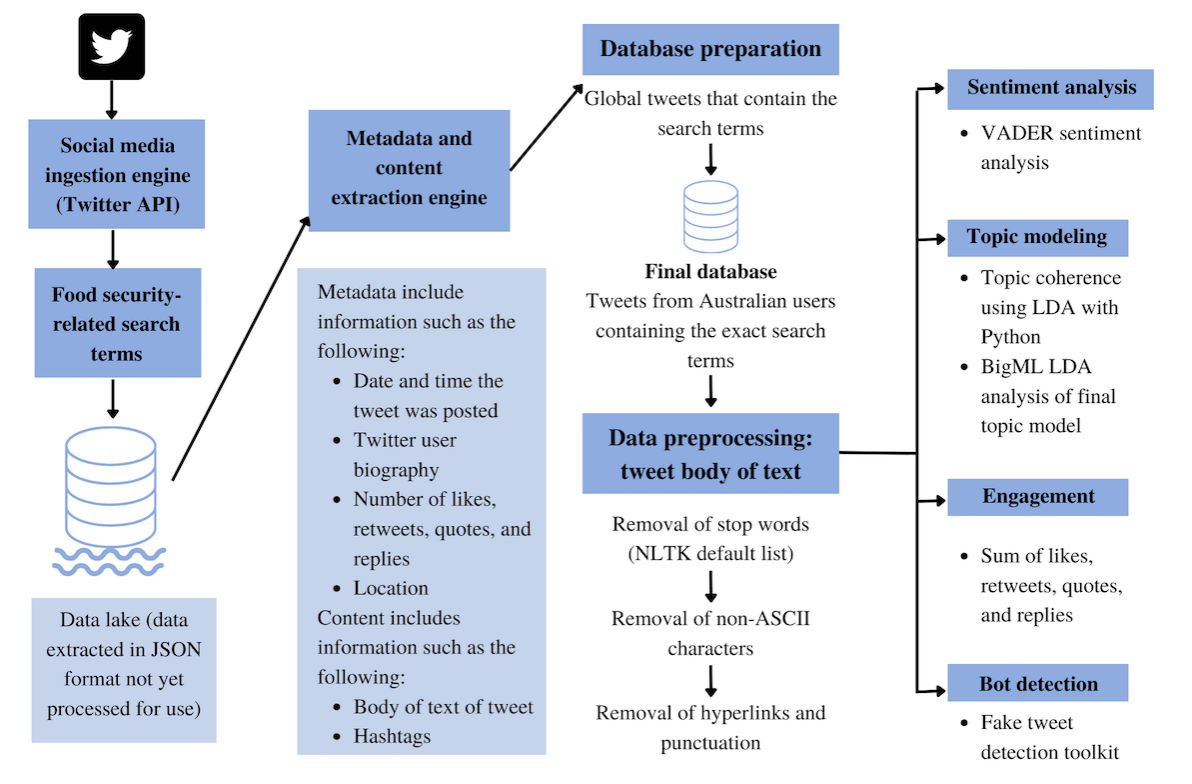
Flow diagram of Twitter data collection, preprocessing, and natural language processing analysis. API: application programming interface; ASCII: American Standard Code for Information Interchange; LDA: latent Dirichlet allocation; NLTK: Natural Language Toolkit; VADER: Valence Aware Dictionary and Sentiment Reasoner.

Data from 2019, 2020, and 2021 were collected using the Twitter API from January 1, 2019, to December 31, 2021 (Figure 1). The Twitter Advanced Search API (rather than scraping) was used to extract all publicly available global original tweets, retweets, and tweet replies that contained at least one of the relevant search terms outlined previously. Another inclusion criterion was English-language tweets. Owing to the rate limits of the API, the data were collected in retrospect over a 1-month period. There were 500 tweets collected in each API call with a wait time of 2 seconds between each call. The process consisted of using the search terms in the “Ingest Tweets” function of the engine to collect up to 500 tweets. These were subsequently written into a JSON file. If there was a next token, the engine would pause for 2 seconds before ingesting another set of tweets and writing another JSON file. This process continued until there was no next token. From the Twitter API, the cross-sectional data from these tweets were processed into a data lake in JSON format, which required further processing for final use.

### Data Processing

Data cleaning and processing were performed using the Python software (Python Software Foundation) [54]. Data were processed from the JSON format (Multimedia Appendix 1) using a metadata and content extraction engine built by the Monash Data Futures Institute to process metadata, such as the date and time the tweet was posted; engagement data, including the number of likes, retweets, quotes, and replies; location of Twitter users; Twitter user biography and verification status (Multimedia Appendix 1); body of text of the tweet; presence of any media, such as photos, videos, or Graphics Interchange Format images; and source from which the tweet was shared. Although global data were collected, the analysis only used data from Twitter accounts from Australia. That is, tweets sent only by Twitter users who had an Australian state or territory listed on their Twitter biography were used in the final database. As geolocation data were not available for all tweets, the location of Twitter users was determined using a filter that identified Australian location names in the text. These location names and common abbreviations (eg, *SYD* is a common abbreviation for Sydney, New South Wales, Australia) were sourced from the Australian Bureau of Statistics [55]. Using only Australian data allowed the authors to gather a more nuanced picture of the discussion on food security that was occurring at the time of more specific events and the state of food security and the COVID-19 pandemic specifically in Australia. The final database also consisted of tweets that contained the exact phrase occurring simultaneously for any 2-word or bigram search term (ie, “food security”) to increase the relevance and specificity of the included tweets.

Data preprocessing for sentiment analysis and topic modeling included the removal of stop words from the body of the tweet text to leave only potentially meaningful words. The Natural Language Toolkit (Team NLTK) default stop word list was used [56]. The American Standard Code for Information Interchange, which contains 128 characters including the numbers 0 to 9, the English letters A to Z, and some special characters, was used to remove all words that were not American Standard Code for Information Interchange characters [57]. Punctuation and hyperlinks were also removed from the tweet text.

With the emergence of the large-scale use of text-generative models (eg, generative pretrained transformer-based content generators), the proliferation of “fake” (or non–human-generated) social media content is ever increasing. Typical misuses of text-generative models include fake news generation, fake product review generation, and spamming or phishing. Eliminating tweets by nonhuman entities (eg, social bots) is a challenge in itself given that 9% to 15% of Twitter accounts are bot accounts (equivalent to almost 48 million Twitter accounts), and these bots generate almost 35% of Twitter content [58]. To eliminate these fake tweets, we used a fake tweet detection toolkit developed by the Monash Data Futures Institute. This toolkit was applied to the original tweet data before preprocessing.

A measure for “engagement” with the Twitter posts was created using the sum of the number of likes, retweets, quotes, and replies each tweet received. The same tweet could occur multiple times across the data set whenever it was retweeted by an eligible Twitter account (ie, from Australia). These retweets were treated as unique tweets in the data set with their own number of likes, quotes, and replies. The tweet source was refined to create an “other” category including categories that accounted for <1% of instances in the data set. This comprised 142 different platforms, including Instagram, LinkedIn, Facebook, and WordPress.

### Data Analysis

#### Sentiment Analysis

Sentiment analysis was conducted using the Valence Aware Dictionary and Sentiment Reasoner (VADER), which is an open-source linguistic rule and lexicon-based sentiment analysis tool [59]. The tool is based on grammatical and syntactical rules that describe word order–sensitive relationships. This includes degree modifiers that affect the intensity of the sentiment of a sentence. That is, “the service is very good” has a higher positive sentiment than “the service is good” because of the addition of “very” [59]. VADER also uses a lexicon with words assigned to a polarity on a scale of –1 (very negative) to +1 (very positive) based on the average polarity score of the words within the lexicon assigned by 10 independent human raters [59]. This lexicon was specifically designed to analyze social media content, including the sentiment of emojis [59].

The lexicon of the VADER sentiment analyzer (ie, the allocation of a sentiment to each non–stop word) was reviewed to ascertain whether there was agreement between the top 100 most frequent non–stop words in the development data set and their assigned sentiment (ie, very positive, positive, negative, very negative, or neutral). After the data were cleaned and preprocessed, the text of each tweet was processed using the VADER sentiment engine. VADER applies the polarity score to each word present in the tweet text to create 5 outputs. All the positive words create a positive score, the negative words create a negative score, and the neutral words create a neutral score [59]. These 3 scores are summed to create a compound score, which is then normalized between –1 (most negative) and +1 (most positive) [59]. The compound score is then classified into sentiment categories: very negative, negative, neutral, positive, or very positive [59], as outlined in Textbox 1.

**Examples of sentiment categories.**

**Example tweet text extracts with corresponding sentiment**

- Example positive tweet (compound sentiment score=0.76): “A big thanks to all those who donated today to our food bank.”
- Example neutral tweet (compound sentiment score=0): “Roughly 25,000 people [in] New Brunswick used food bank services including soup kitchens.”
- Example negative tweet (compound sentiment score=–0.67): “Economic growth sub par years high unemployment casualisation record underemployment wages stagnant food insecurity house prices dropping LNP [Liberal National Party] blame next Labor Govt #auspol.”

#### Topic Modeling

The tweet text was used to develop themes or topics through the process of topic modeling using latent Dirichlet allocation (LDA). LDA is a probabilistic algorithm that groups similar text-based data that commonly occur together within a data set into themes [7,33]. As LDA topic modeling is an unsupervised machine learning process, the model created is based on the data themselves and the relationships found within the words present in the textual data. Given its unsupervised nature, LDA topic modeling does not specify the number of topics that are most appropriate or representative of the themes for the data set and, therefore, requires the specification of the number of topics to be used in the model. Consequently, we used a measure known as coherence to help determine the most appropriate number of topics to include. Coherence measures have been shown to correspond well to a human interpretation of the topics from a data set [60]. Topic coherence can provide a score for a single topic by measuring the degree to which the high-scoring words within that topic are semantically similar to each other [61]. This coherence score is then used to determine whether a set number of topics for that data set are semantically interpretable rather than being related only because of statistical inference [61].

**Figure 2:**
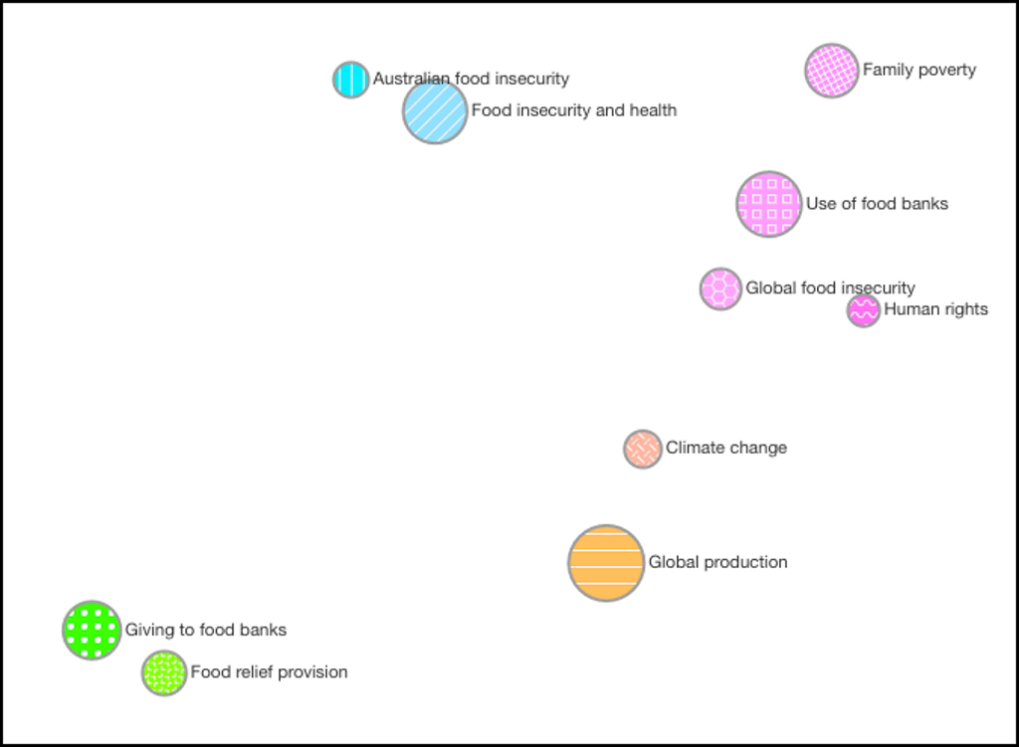
Topic model distribution of Twitter food security data as visualized on the topic map from BigML (BigML, Inc). The size of the circle represents the probability of that topic being discussed within the data set, and the position of the circles represents the thematic closeness of the topics. There are no axes to consider when interpreting this figure.

For this study, we used the Gensim (RARE Technologies Ltd) implementation of LDA topic coherence [62], which is an implementation of a 4-stage topic coherence specified by Röder et al [63]. The results of coherence testing revealed that the models with the highest coherence scores were for a topic model with 19 topics (coherence score=0.481) and 10 topics (coherence score=0.478). To determine semantic coherence at the human level, a model including the 10 and 19 topics was created using BigML (BigML, Inc). Manual coherence testing involved visually inspecting the models created through BigML, including the spread of the topics, coherence of the top 10 terms within each topic, and distinction among the different sets of top-10 words within each topic. BigML displays the topics in a topic map (Figure 2) by plotting topics as circles, with the size of the circle representing the topic probability [64]. The position of the topics in the 2D plane of the map is defined by the thematic closeness among the different topics [64]. The topic model with 19 topics (Figure S1 in Multimedia Appendix 2) consisted of topics with a high crossover of key terms and little semantic differentiation. From this manual coherence testing, it was determined that 10 topics (Figure 2) had the greatest semantic coherence.

The final topic model was created using the BigML web machine learning platform, which uses an implementation of LDA [64]. The following settings were used for the topic modeling: 10 topics (as determined during coherence testing); 10 top terms per topic; stemming of words, which reduces words to their word stem (eg, the words “agriculture” and “agricultural” would be grouped together and treated as 1 word); and use of bigrams, which allowed for the inclusion of 2-word phrases as 1 term (eg, food security). The batch topic distribution function in BigML was then used to determine, for each tweet, the probability that it discussed each of the 10 topics [64]. Each tweet within the data set had a probability value of that tweet being categorized into each of the 10 topics. The topic with the highest probability was then assigned as the topic for that tweet. For the final topic model, the names were based on the top 10 terms within each topic. In total, 3 authors (AM, LB, and TAM) independently determined names before coming together to triangulate and finalize the most appropriate topic names.

#### Statistical Testing

Normality testing was conducted using the Kolmogorov-Smirnov test, histograms, and *Q*-*Q* plots using the Python software SciPy statistics program [65]. The data were found to be not normally distributed, and therefore, median and percentiles and nonparametric tests were used where applicable. The Kruskal-Wallis test was conducted to explore differences in engagement for tweets with different topic and sentiment classifications. Post hoc Dunn tests were used when the Kruskal-Wallis test was significant at *P*<.05. The chi-square test for independence was used to determine the differences between categorical variables.

### Visualization and Interpretation

The Python library *matplotlib* was used to visualize the data [66]. This included observing the evolution of sentiment and topics across time, which involved plotting changes in sentiment and topic across the years, months, and quarters. To help with the interpretation of sentiment and topic evolution across time, the COVID-19 pandemic situation in Australia at the time of tweet collection was considered. The COVID-19 pandemic and related lockdowns had substantial effects on food security in Australia and worldwide [67]. Therefore, it is important to consider the effect of the pandemic when assessing tweets related to food security during this period. To explore any associations with COVID-19 lockdowns in Australia, the sentiment and topic evolution graphs were plotted with shading for times when a state or territory of Australia was in a COVID-19 lockdown [68,69]. If that month had more than a week of lockdowns in total across the Australian states and territories, it was shaded in gray. Given that the data included tweets from users from anywhere in Australia, it was decided to shade any lockdowns occurring in Australia even when only 1 state or territory had an active lockdown. The lockdowns differed in their level of restrictions, with some states or territories imposing travel limits (ie, not being permitted to travel >5 km from the place of residence) during some periods. The lockdowns primarily imposed restrictions on retail businesses that people were permitted to access. The businesses to which access was permitted were generally only supermarkets, take-away food restaurants, and pharmacies, and people were also able to undertake other essential travel, such as seeking medical treatment and going to work when it was deemed essential to attend the worksite in person.

In addition, Australia introduced COVID-19 support payments for those who were unable to work or lost their jobs because of the COVID-19 pandemic [70]. The date when this was introduced in March 2020 and when it was removed in March 2021 [70] were also plotted on the sentiment and topic evolution graphs. Alongside these COVID-19 food security–related events in Australia, key news events related to food security and the topics identified through topic modeling were tracked for each month for qualitative discussion within the results [71]. During manual exploration of the tweets, it was evident that the issues discussed included topics broader than the state of food security in Australia alone, which was also highlighted in the topic modeling. Therefore, it was decided to examine news headlines of global as well as Australian-based events related to food security and the topics from topic modeling [71]. This was a broad comparison with news headlines from one source and, therefore, explored associations with food security events and the potential usefulness of examining news headlines in this way but did not determine the specific reasons behind the sentiment and topic trends.

### Ethical Considerations

The data for this study were collected through public Twitter profiles, and we adhered to the privacy policies, terms of use, and terms and conditions of Twitter; we aggregated only anonymized data without displaying user identification. Ethics approval for this study was granted by the Monash University Human Research Ethics Committee (approval 27376).

## Results

### Tweet Descriptives

After filtering the Twitter data by search terms across the 3-year period, there were 38,070 tweets from 14,880 unique Australia-based Twitter users. Of the Twitter users included in our study, most were not verified users (36,302/38,070, 95.36%; Table 1), which indicates fewer organizational accounts or well-known figures discussing the issues. There was a larger proportion of tweets in 2020 than in 2019 and 2021, with 2021 having the second highest number of tweets and 2019 having the lowest number of tweets. Retweets were the most common tweet type, accounting for >70% (28,062/38,070, 73.71%) of the sample and remaining the most common across all years of the study period. The tweets generally consisted of text only (34,230/38,070, 89.91%), which is most appropriate for text-based NLP. This was consistent in 2020 and 2021, but in 2019, there were a higher number of tweets with photos. The fake tweet detection toolkit estimated that a large majority of tweets (34,895/38,070, 91.66%) did not come from suspected bot accounts; however, the proportion of tweets from suspected bot accounts increased across the years from 6.09% (643/10,562) in 2019 to 11.3% (1379/12,206) in 2021.

**Table 1:** Food security tweet descriptive data over the study period (2019 to 2021; N=38,070).

		Total, n (%)	2019 (n=10,562), n (%)	2020 (n=15,302), n (%)	2021 (n=12,206), n (%)
**Tweet type^a^**					
	Original tweet	4121 (10.82)	1218 (11.53)	1612 (10.53)	1291 (10.58)
	Retweet	28,062 (73.71)	7800 (73.85)	11,268 (73.64)	8994 (73.69)
	Reply	3918 (10.29)	954 (9.03)	1595 (10.42)	1369 (11.22)
	Quote	1969 (5.17)	590 (5.59)	827 (5.4)	552 (4.52)
**Tweet format^a^**					
	Text only	34,230 (89.91)	9199 (87.1)	13,851 (90.52)	11,180 (91.59)
	Photo	3747 (9.84)	1336 (12.65)	1416 (9.25)	995 (8.15)
	Animated GIF	66 (0.17)	17 (0.16)	33 (0.22)	16 (0.13)
	Video	2 (0.01)	10 (0.09)	2 (0.01)	15 (0.1)
**Twitter user verification^a^**					
	Verified	1768 (4.64)	427 (4.04)	822 (5.37)	519 (4.25)
	Not verified	36,302 (95.36)	10,135 (95.96)	14,480 (94.63)	11,687 (95.75)
**Authenticity^a^**					
	Suspected bot account	3175 (8.34)	643 (6.09)	1153 (7.53)	1379 (11.3)
	Not suspected bot account	34,895 (91.66)	9919 (93.91)	14,149 (92.47)	10,827 (88.7)

^a^Chi-square test of independence significant at *P*<.001 for differences between the years.

### Sentiment Analysis and Sentiment Evolution

#### Overview

The most predominant sentiment among the data set was positive (14,966/38,070, 39.31%), followed by negative sentiment (11,638/38,070, 30.57%; Table S1 in Multimedia Appendix 3). The extreme ends of the sentiment scale, that is, very positive and very negative, were the least common. There was a significant difference (*P*<.001; Table S1 in Multimedia Appendix 3) in the sentiment proportions across the 3-year period, with negative sentiment being slightly more predominant in 2019 and neutral and positive sentiment being slightly more predominant in 2021.

#### Sentiment Evolution in Relation to News Headlines

**Figure 3:**
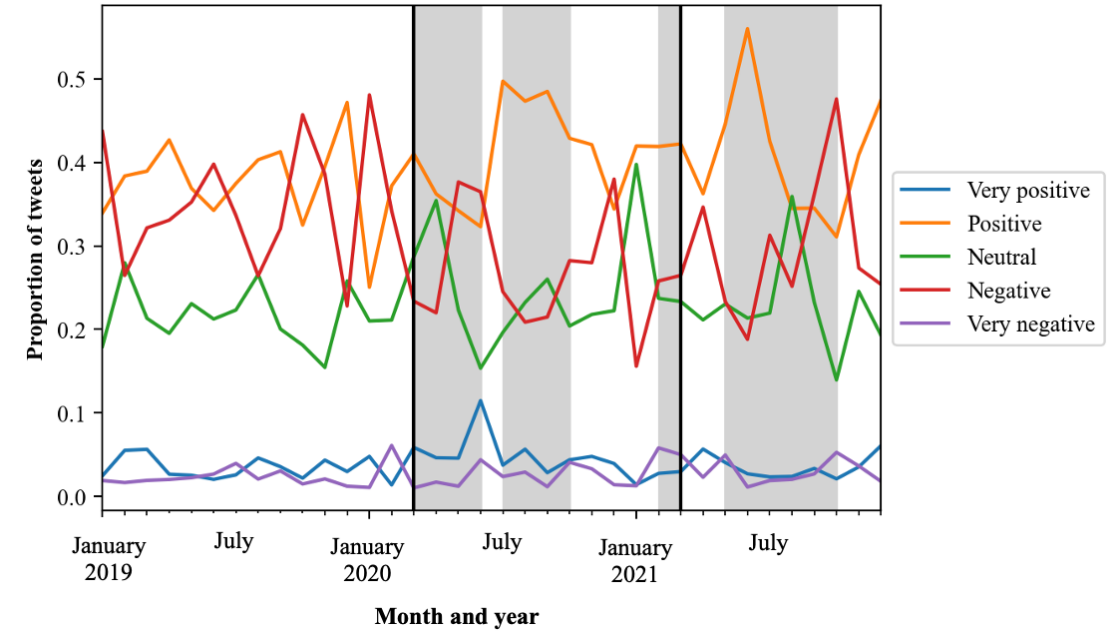
Proportion of sentiment by month for Twitter food security data. The black lines represent the start and end of COVID-19 support payments by the Australian Government; the gray-shaded sections represent periods when there was a COVID-19 lockdown in an Australian state or territory that lasted >1 week in total within the month.

When examining sentiment evolution by month (Figure 3), positive sentiment was high in July 2020, which was dominated by events related to COVID-19 in Australia. In addition, positive sentiment was high in June 2021, when the Australian Government announced a new COVID-19 disaster payment to replace the earlier support payments for people without work. This month, the World Food Programme also warned of famine across Africa arising from the COVID-19 pandemic. Negative sentiment was highest in October 2019 and January 2020, a period that was characterized by the Australian bushfire season dubbed “Black Summer” in which many homes were destroyed and families were displaced and required emergency food relief. Another period in which positive sentiment was high was October 2021, which was also when Madagascar faced a food crisis and, in Australia, COVID-19 lockdown restrictions were beginning to ease after the longest run of lockdowns across the country. Very positive sentiment was high in June 2020, which was a month with no COVID-19 lockdowns. From July 2020 to September 2020, positive sentiment was high despite the COVID-19 lockdowns across Australia. During the subsequent lockdowns in 2021, positive sentiment continued to be dominant despite public discontent throughout the lockdown periods. Neutral sentiment rarely had the highest proportion, only being greater than positive or negative sentiment in August 2021, which demonstrates that the conversation was generally polarized across the years.

### Topic Modeling

The topic model distribution with the highest topic coherence score comprised 10 topics. “Global production” considered food production and agriculture and was the most predominant topic in the data set with the highest probability (Table S2 in Multimedia Appendix 3 and Figure 2). “Global production” was clustered on its own, and its closest relationship was to “Climate change,” which also discussed issues regarding agriculture because of its focus on the environment (Figure 2). “Food insecurity and health” was the second most probable topic within the data set, characterized food insecurity as a public health issue, and was clustered with “Australian food insecurity.” There was a cluster of 4 topics that included “Use of food banks,” “Family poverty,” “Global food insecurity,” and “Human rights.” Finally, a cluster of 2 topics with the furthest distance discussed “Giving to food banks,” including donation and volunteering, and “Food relief provision” at the national level.

### Topic Evolution Across Time

#### Overview

**Figure 4:**
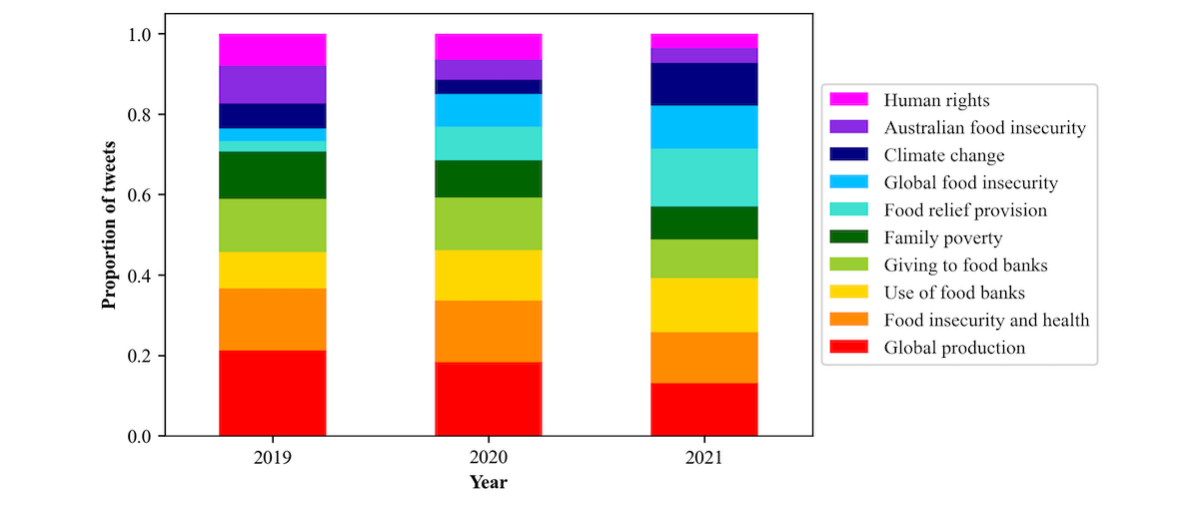
Topic evolution by year based on the proportion of food security tweets assigned to each topic created through topic modeling.

Within the data set, a probability value was calculated for individual tweets across all 10 topics. When examining the topic with the highest probability across the years, most tweets in both 2019 and 2020 discussed “Global production” (2246/10,562, 21.26% and 2810/15,302, 18.36%, respectively; Table S2 in Multimedia Appendix 3 and Figure 4). In 2021, “Food relief provision” accounted for the highest proportion (1762/12,206, 14.44%) of tweet topics, followed by “Use of food banks” (1641/12,206, 13.44%).

**Figure 5:**
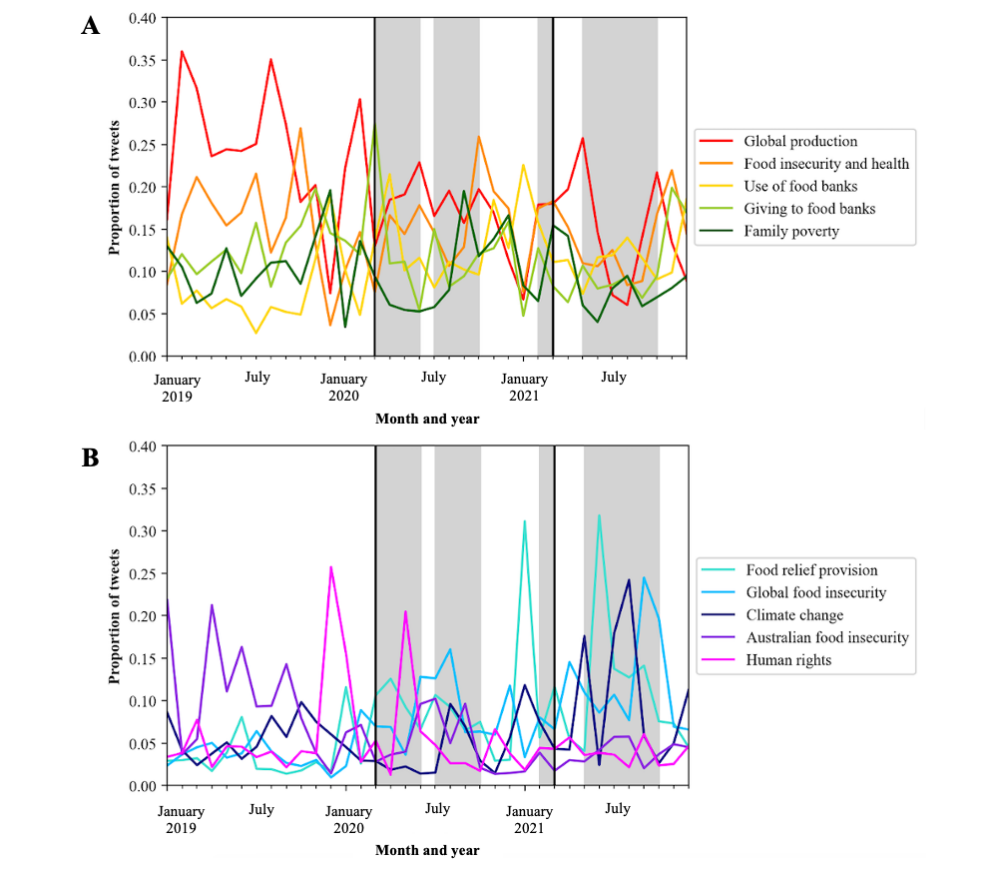
Proportion of highest-probability topics created through topic modeling by month. (A) Topics 1 to 5; (B) Topics 6 to 10. The black lines represent the start and end of COVID-19 support payments by the Australian Government; the gray-shaded sections represent periods in which there was a COVID-19 lockdown in an Australian state or territory that lasted >1 week in total within the month.

When examining the evolution of topic discussion across individual months, “Global production” had the highest probability for 50% (18/36) of the months, and “Food insecurity and health” had the second highest probability for 17% (6/36; Figure 5) of the months. Topic evolution by quarter (Figure S2 in Multimedia Appendix 2 and Table S3 in Multimedia Appendix 3) was not as nuanced, and therefore, the changes by month were used for further exploration.

#### Topic Evolution in Relation to News Headlines

The proportion of predominant topics among the tweets varied across the months (Figure 5). “Global production” remained a largely discussed topic across time, with dominant periods in February 2019, August 2019, and February 2020, with February 2020 including an announcement that Europe had experienced its warmest January on record and the beginning of the rise of COVID-19 outbreaks and deaths across the world. “Food relief provision” also had high periods in January 2021 and June 2021, with a high proportion of positive sentiment also in June 2021. In June 2021, the World Food Programme warned of famine across Africa owing to the COVID-19 pandemic. In addition, the Australian Government announced a new disaster payment that replaced earlier support payments for casual workers who lost work because of the lockdowns and had no support payments during such times.

Other notable high-proportion periods of topics by month included “Giving to food banks” in March 2020, which corresponded to the beginning of the COVID-19 lockdowns across Australia. “Family poverty” was at its highest proportion in December 2019 during the Australian bushfire season (dubbed “Black Summer,” in which many homes were destroyed and families were displaced) and in September 2020, when Australia officially entered a recession for the first time since 1991. Despite these events, positive sentiment was high in September 2020 (Figure 3). “Global food insecurity” was at its highest proportion in September 2021, which was when there were news headlines of war in Tigray, Ethiopia, with people facing severe food insecurity, and a United Nations Children’s Fund report was released that highlighted that, in 91 countries, most infants experience malnourishment and food insecurity [72]. “Climate change” was at its highest proportion in August 2021 during a heat wave and ongoing wildfires across Europe. The topic of “Human rights” was at its highest proportion in December 2019, with many civilians fleeing Syria during the Syrian civil war, and also in May 2020. Topics specifically related to the prevalence of food insecurity, poverty, and food relief in Australia were not consistently more prominent during the COVID-19 pandemic than during the prepandemic period.

### Sentiment and Topic

**Figure 6:**
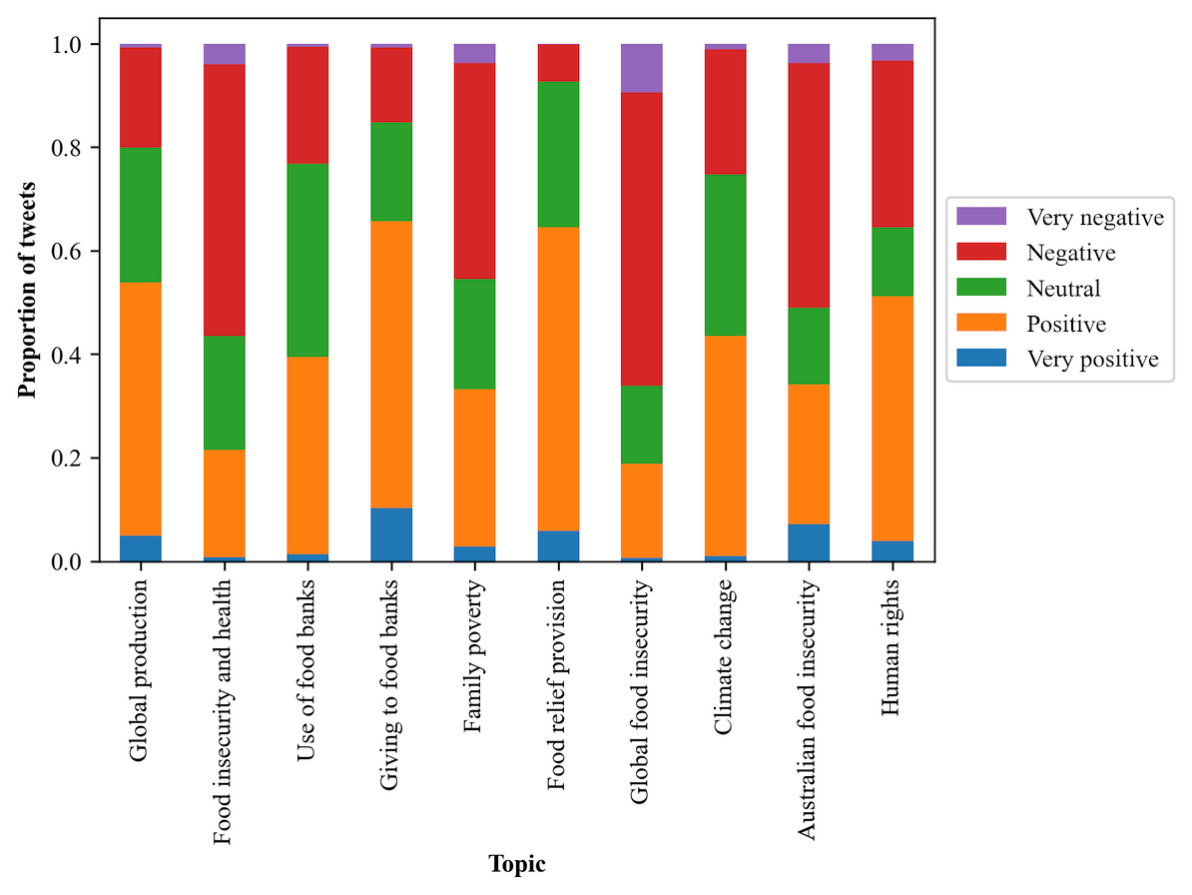
Proportion of food security tweets in sentiment categories by topic created through topic modeling.

There was a significant difference between the proportion of sentiment categories for each topic (*P*<.001; Table S4 in Multimedia Appendix 3), as shown in Figure 6.

“Global production” of food was discussed with a generally positive sentiment (3262/6656, 49.01%). “Food insecurity and health” and “Family poverty” were more negative in their sentiment (2885/5487, 52.58% and 1530/3670, 41.69%, respectively). “Use of food banks” by individuals had the highest proportion of neutral sentiment (1692/4534, 37.32%) across the topics and was otherwise largely positive (1727/4534, 38.09%). “Giving to food banks,” which centered on donations and charitable support, had the highest proportion of positive sentiment (2526/4561, 55.38% positive and 467/4561, 10.24% very positive). The topic with the highest proportion of positive sentiment (1944/3318, 58.59%) was “Food relief provision.” Both “Global food insecurity” and “Australian food insecurity,” which discussed food insecurity prevalence, were predominantly negative (1620/2859, 56.66% and 1052/2223, 47.32%, respectively), and “Global food insecurity” had the highest proportion of negative and very negative sentiment (270/2859, 9.44%). “Climate change” in relation to food security was discussed with generally positive (1067/2506, 42.58%) or neutral (780/2506, 31.13%) sentiment. When discussing “Human rights” topics such as access to shelter, food, and water, a positive sentiment was generally expressed (1067/2256, 47.3%). Topics with a predominantly negative sentiment contained more negative key terms such as “insecurity” and “poverty,” whereas some predominantly positive topics had key terms with positive sentiment such as “support” and “help” (Table S2 in Multimedia Appendix 3).

### Tweet Engagement

The overall engagement score for the tweets was significantly higher in 2021 than in 2019 and 2020 (*P*<.001; Table 2). In the overall sample, very negative and negative tweets received substantially higher engagement (Table 2). When comparing across years, this was also true for 2019 and 2020; however, engagement was significantly higher for neutral tweets in 2021 (*P*<.001). Despite “Global production” being the most frequently discussed topic, engagement was significantly lower than for other topics overall and remained low across the years (*P*<.001). Overall, “Climate change,” “Human rights,” and “Family poverty” received the most engagement (median 142, 126, and 111, respectively). In 2019, only “Human rights” and “Family poverty” remained the topics that received the highest level of engagement. In 2020, the topic with the highest level of engagement was also “Human rights,” whereas in 2021, this topic had a much lower engagement rate (median 381 and 4, respectively). “Climate change” engagement was at its highest level in 2021 (median 200). Topics that received consistently lower engagement across the years were “Global production,” “Food insecurity and health,” and “Giving to food banks.” There was no clear relationship between the predominant sentiment of a topic and engagement with that topic, with 2 of the top 3 topics with the highest engagement having an overall positive sentiment and the third having a negative sentiment.

**Table 2:** Twitter engagement of food security tweets with different highest-probability topics created through topic modeling and sentiment analysis^a^ (superscripted letters denote statistical significance).

				Engagement total, median (IQR)		Engagement in 2019 (n=10,562), median (IQR)		Engagement in 2020 (n=15,302), median (IQR)		Engagement in 2021 (n=12,206), median (IQR)
Engagement (sum of likes, replies, quotes, and retweets)				11 (2-165)		10 (2-141)^b^		11 (2-123)^b^		15 (3-270)^c^
**Sentiment**										
	Very negative			18 (2-205)^d^		20.5 (4-560)^b^		38 (4-126)^b^		10 (1-209)^b,e^
	Negative			22 (3-238)^d^		25 (4-244)^b^		28 (4-381)^b^		13.5 (2-128)^b^
	Neutral			11 (2-304)^f^		7 (2-86)^c^		9 (2-99)^c^		34 (3-930)^c^
	Positive			9 (2-100)^g^		7 (2-102)^c^		7 (2-46)^h^		13 (3-247)^h^
	Very positive			6 (2-22)^i^		4 (2-9)^h^		9 (3-94)^c,h^		5 (2-14)^e^
**Topics and predominant sentiment**										
	**Global production**									
		Positive	5 (2-14)^d^		4 (1-10)^b^		6 (2-18)^b^		5 (2-15)^b^	
	**Food insecurity and health**									
		Negative	7 (2-25)^f^		7 (2-19)^c^		8 (2-34)^c^		6 (2-22)^b^	
	**Use of food banks**									
		Positive and neutral	37 (3-501)^g^		85 (5-1715)^e,h^		25 (2-361)^e,h^		48 (3-501)^c^	
	**Giving to food banks**									
		Positive	8 (2-79)^i^		8 (2-58)^j,k^		8 (2-73)^c^		7 (2-131)^h^	
	**Family poverty**									
		Negative	111 (5-2167)^l^		285 (11-4033)^m^		125 (4-2214)^j,n^		42 (3-1288)^c^	
	**Food relief provision**									
		Positive	12 (3-171)^o^		4 (1-10)^b,c^		6 (2-18)^b^		51 (5-930)^c^	
	**Global food insecurity**									
		Negative	13 (2-129)^i,o^		6 (1-21)^b,c,j^		38 (4-238)^h^		6 (1-86)^b^	
	**Climate change**									
		Positive	142 (14-581)^l^		78 (5-244)^h,n^		25 (3-345)^e,h,j,m^		200 (43-609)^e^	
	**Australian food insecurity**									
		Negative	19 (3-141)^o^		23 (2-141)^k,n^		18 (3-121)^e,m^		11 (3-247)^h^	
	**Human rights**									
		Positive	126 (3-1053)^g,l^		1053 (5-1053)^e,m^		381 (3-8509)^n^		4 (1-127)^b,h^	

^a^Predominant sentiment refers to the sentiment with the highest proportion for each topic, as shown in Figure 6 and Table S4 in Multimedia Appendix 3. *P*<.001 Kruskal-Wallis test for differences between topic and year and differences between sentiment categories.

^b-o^Values within topic overall, sentiment overall, and topic and sentiment by each year with different superscript letters are significantly different from each other using the post hoc Dunn test and Bonferroni correction.

## Discussion

### Principal Results

This study used the NLP techniques of sentiment analysis and topic modeling to explore the conversation around food security on Twitter in Australia. The key findings indicate that the overall sentiment of the tweets related to food security was positive, although this varied when assessed by month across the 3-year study period. Positive sentiment remained higher than other sentiment categories during the COVID-19 lockdown periods in Australia. Extremes of sentiment (ie, very negative and very positive) were not common, and neutral sentiment remained lower than both positive and negative sentiment throughout the study period except for August 2021. A topic model with 10 topics related to food security was created based on high topic coherence. The most predominant topic, “Global production,” was related to food production and agriculture, which clustered semantically on its own, with the closest related topic being “Climate change.” There were several topics related to food relief and food banks, with different focuses related to public health, volunteering and donation, government support of food banks, and use of food banks by families. When comparing predominant sentiment in the topics, “Giving to food banks,” which focused on support and donation to food banks, had the highest proportion of tweets with positive sentiment, and “Global food insecurity,” which refers to the prevalence of food insecurity worldwide, had the highest proportion of tweets with negative sentiment. Negative tweets received substantially higher engagement in 2019 and 2020 than in 2021. Despite being the most frequent topic, “Global production” received substantially lower engagement. There was no clear relationship between the predominant sentiment of topics and the engagement rate.

### Comparison With Prior Work

Infodemiology involves a specific way of developing knowledge through web-based data sources and can explore discussions and potential influences on health; however, its application in the area of food security as a public health issue is still emerging. Previous studies have used a variety of NLP techniques as part of an infodemiological process to explore different areas of food security for different purposes; however, they have not combined sentiment and topic analysis across time and in relation to social media engagement, as in this study. Studies have used NLP to predict food deserts using the sentiment and nutritional value of the foods mentioned on social media within different geographic regions [73] and to summarize the academic literature on community gardens [74]. Research similar to this study includes a study by Mayasari et al [75] that used Google Trends to explore food security and dietary and lifestyle behaviors during the COVID-19 pandemic. Our study found an increase in the popularity of food security conversations at the beginning of the COVID-19 pandemic, with similar findings related to frequency of discussion to those of Mayasari et al [75], who also found that Australia was among the top countries in search frequency regarding food security. Similarly, Martin et al [76] used topic modeling of tweets related to food security during the COVID-19 pandemic and found an increase in posts on food security, particularly related to food banks and food relief. Martin et al [76] also found topics similar to those in our study, with food assistance, needs, and resources found to be the most dominant topic category, and these topics similarly discussed free food, donation, government assistance, food systems, and food banks. Martin et al [76] also highlighted the topic of emergency preparedness, which covered individual family crises, COVID-19–related food insecurity, and emergency aid in Tigray, all of which were discussed in tweets in our study. The time-series topic analysis by Benites-Lazaro et al [77] highlighted discussions on ethanol production (the topic of the study) and food security dominated by government bodies in comparison with nongovernment and media and peaking from 2007 to 2009, which aligned with the world food crisis. *Government* was a keyword in 2 topics in this study: “Food relief provision” and “Human rights”; however, as Twitter was the data source, there would not likely be the same amount of information coming from government sources as that in the study by Benites-Lazaro et al [77], which used government documents as one of its data sources.

Using a manual analysis approach to infodemiology, research has also examined Facebook posts in community groups in the Pacific Northwest of the United States and found that food assistance and free meals were the most commonly discussed topics in relation to food security [78]. Although conducting only manual analysis, Nguyen et al [78] also found that Facebook posts about community gratitude and those that incorporated culture received more engagement than other posts. These previous studies and our study highlight the potential for computational analysis of infodemiology (eg, sentiment analysis and topic modeling), which allows for the exploration of a broader range of information to gather insights into food security and public health.

Sentiment analysis techniques have been used to achieve various goals in the public health arena of food security. On a broad scale, sentiment analysis has been used to assess the academic literature on food security worldwide [79]. Masih et al [79] found that predictors of positive sentiment included empowerment, farming, and certain government policies and interventions, whereas predictors of negative sentiment included climate change and other government policies and interventions [79]. In Australia, climate change has presented a major challenge and is a predictor of negative sentiment and dissatisfaction with the government’s response to climate change and natural disasters [79]. In comparison, our study found that discussions on global production and farming had a positive sentiment and that the topic of climate change overall also had a positive rather than negative sentiment. In addition, our study found that the government was primarily discussed in relation to food relief and food security rather than in relation to climate change or agriculture. A study with a different focus used sentiment analysis to predict sentiment on agriculture by farming communities on Twitter given that crop yields are a measure of food security in different geographical areas [80]. Sentiment analysis has found that, when discussing staple foods in Indonesia as a measure of food security, price volatility and the inability to purchase staple foods at current prices are predictors of negative sentiment [81].

Although this study examined food security more broadly, sentiment analysis has also been used to investigate specific areas of food security. For example, a study assessing the sentiment of web-based conversations specifically related to local food and food banks found that the net sentiment was negative [82]. In contrast, the Twitter posts in this study classified in topics related to food banks were largely positive. This is likely due to the type of discussion in the study by Jung et al [82] being dominated by negative words such as “struggle,” “difficult,” and “desperate,” whereas this study discussed more positive aspects, such as donations, support of food banks, and having access to these services. In addition, Scott et al [83] examined the Supplemental Nutrition Assistance Program (SNAP) in the United States. These researchers found that news articles discussing the SNAP with extreme right media bias were more likely to score on either side of extreme sentiment [83]. When events such as budget cuts to the SNAP occurred, news articles were more likely to have negative sentiment [83], highlighting the potential of sentiment analysis as an efficient indicator of the state of a topic at different time points or during different periods. However, on its own, sentiment analysis can provide only limited detail on text-based data, and therefore, other data analysis methods such as topic modeling can be used to capture further meaning.

The topic modeling findings of this study can be used in data triangulation with data related to food security from other sources, including sources that are not social media. Previous research has used topic modeling of YouTube and newspaper data and found some consistency between the regions discussing food security and household survey data on the food security risk of these same regions, potentially serving as early warning signals for at-risk areas [84]. Our study found that the topics in food security social media data reflected all the dimensions of food security as defined by the Food and Agriculture Organization [3] and Clapp et al [39] to different extents. The topics most commonly covered the dimensions of access and stability, whereas availability, sustainability, and use of nutrition and agency were less commonly the focus of the themes. Despite the tweets being from Australian users only, there was a range of tweets covering food security issues worldwide from both the public health and economic perspectives. This included food insecurity prevalence, with the topic “Global food insecurity” increasing in number of tweets across the 3-year period and being predominantly negative in sentiment. These findings align with the increase in people experiencing food insecurity worldwide during this time and the associated health issues of undernourishment, particularly during the COVID-19 pandemic [40]. The topic of “Global production” covered worldwide issues related to agriculture and food production and had the greatest probability of being discussed despite having low engagement. This low level of engagement may indicate the distance between the issue being on a global scale and the individual feeling that they are not personally responsible, therefore making them less likely to engage with the topic. Other research findings highlighted issues regarding production, with food imports and exports slowing and sometimes even stopping throughout the pandemic because of factors such as shortages of labor in agriculture and food production and lack of agricultural supplies having major effects on the food supply chain worldwide [85]. Apart from the COVID-19 pandemic, climate change was also a global threat to food security identified in the topic model. Previous research has demonstrated that adverse weather events, including droughts, flooding, and cyclones, have a major impact on agriculture and food production, with reduced crop yields and subsequent instability in food prices [86,87]. However, sentiment analysis of the topic of “Climate change” showed that tweets on this topic were primarily positive, indicating that the discussion on the effects of climate change on food security was framed more positively and potentially more toward opportunities or solutions in this area.

Of the topics covered in our data set of tweets, 3 were clearly related to food security issues specific to Australia, which was evident in the inclusion of Australia-specific key terms (eg, *Australia* and *auspol*). “Food insecurity and health” was largely related to food security in the wake of the COVID-19 pandemic in Australia. This topic aligned with the response to COVID-19 in Australia, which included lockdowns across individual states and territories from March 2020 to October 2021. Although these lockdowns kept case numbers relatively low, they resulted in substantial income loss [88]. To address income loss because of the closure of many industries, the Australian Government introduced a wage subsidy scheme and increase to welfare [88]. Although this is estimated to have reduced total job losses [89], there was an increase in people experiencing food insecurity in Australia up to an estimated 19% to 26% [90,91]. People receiving these payments were up to 3.5 times more likely to experience food insecurity than employed individuals [91]. In this study, the topic “Australian food insecurity” included discussion of a call to action for the Australian Government to address the situation of people experiencing food insecurity marked by a negative sentiment, which may indicate dissatisfaction with the government action or an increased prevalence of experiencing food insecurity and subsequent health issues.

Data collected during the COVID-19 pandemic highlighted an increased reliance on food relief during this period in Australia [92]. This increase was reflected in the topics related to food security in the social media data collected in this study, with 3 different topics specifically related to aspects of food relief and food banks. The predominant response to food insecurity in Australia remains the provision of food relief [48], and this was evident throughout the years of the COVID-19 pandemic [93]. However, in this study, topics related to food insecurity prevalence and food relief within Australia were not consistently more prevalent throughout the pandemic than they were the year before the pandemic, suggesting that these issues were of key interest before the pandemic. In addition, the topic model highlighted key groups that were accessing food relief (eg, students) in the topic “Use of food banks.” In Australia, through the demand for emergency food relief during the pandemic, students, particularly international students, were highlighted as a group of people experiencing food insecurity and the related mental health consequences for the first time [93]. This study also highlights key events that may have influenced how the topic area of food security was reported. This includes high proportions of negative sentiment and the topic of “Family poverty” during the bushfires in the Australian summer of 2019 to 2020 (Black Summer). This bushfire season saw many homes destroyed and people displaced as well as loss of livestock and disruption of agricultural land [94]. Although it is not certain that this event influenced the negative sentiment, topic modeling and sentiment analysis can help explore issues that may be driving conversations across time.

### Suggestions for Future Research

Future research on food security could focus on furthering infodemiology techniques through interdisciplinary teams that can use NLP techniques in a health-specific context. This study highlighted the value of the information interpretation techniques of topic modeling and sentiment analysis, which can be used to interpret social media data in the areas of food security. The topics highlighted in the data set had some alignment with events and topics of interest in the food security domain identified through different sources. However, future use of broader data sets including information such as stakeholder opinions could enhance the understanding of associations among sentiment, topic, and news events. The importance of a topic-specific lexicon created by domain experts to gather relevant information was apparent, with other research bodies working toward a comprehensive lexicon in the food security domain [95]. Future research should use the techniques applied in this study as one step within broader infodemiology and infoveillance efforts in public health areas such as food security. Infoveillance could be used to track events that could affect food security at the national or global level, such as climate change, food production, food supply, and government policies related to food security. These techniques could also include using multiple platforms of information—from news articles to different social media platforms and using search terms beyond hashtags—to capture a wider understanding. Infoveillance techniques also have the potential to track trends in the prevalence of food insecurity, thereby enabling the public health sector to improve some of the major effects of food insecurity on health status in a more proactive way by detecting early warning signals [84]. Currently, the prevalence of food insecurity is not completely understood or able to be tracked because of the difficulty in obtaining data and the use of different tools that do not measure all the dimensions of food security [96,97]. This research highlights that, although some topics related to food insecurity are commonly discussed on Twitter, they do not necessarily receive a great deal of engagement from other users. To translate these findings into action, there is a need to engage and connect those talking about these different areas of food security to create collective action.

### Limitations

This study has several limitations that should be acknowledged. First, it used Twitter data as the only social media source. Although Twitter is the most commonly used source of information for infodemiology studies [26], there are limitations to its use as the only source. Individuals who use Twitter have been found to be younger than the general population, more likely to be male, and generally more educated and politically attentive than nonusers of social media [98]. Therefore, the users and the information they post are not necessarily representative of the viewpoints of the wider population. A further limitation of using Twitter could be that it has recently experienced a decline in popularity [99]; however, the study collected the data before this decline. Second, the study used VADER sentiment analysis, which lacks the ability to apply context. VADER cannot consider the context of the words and, therefore, cannot consider the potentially different meanings of words within the context of nutrition and food security. Third, in relation to the examination of engagement, tweets that were posted toward the end of data collection in 2021 had less time to receive engagement on Twitter and, therefore, may not have reached their full potential for engagement. However, because of the fast-paced nature of Twitter, most engagement is likely to occur soon after the tweet is posted [100]. Fourth, inferences about the association between news articles covering key events and different sentiment and topic occurrences are not certain. These events are not necessarily the reason for these occurrences as it cannot be determined among the large data set of this study whether these specific events were the most discussed during these periods.

### Conclusions

There is potential for the use of NLP techniques to explore social media data to further understand complex areas of public health such as food security. In this study, we demonstrated the value of sentiment analysis and topic modeling in exploring changes in sentiment and key topics discussed in Australia. Topic modeling highlighted the focus on food relief and food banks in the context of Australia and on broader food security themes of global production and supply of food and the effect of climate change on food security. The food security data overall had a slightly more positive sentiment; however, posts with negative sentiment received higher engagement, suggesting the tone of discussion in this topic area that may gather the most attention. Key topics of interest and sentiment evolved throughout the 3-year period, including during the COVID-19 pandemic; however, positive sentiment when discussing food security remained high even throughout lockdowns and subsequent food security crises in Australia. However, because of the discrepancies in associations among sentiment, topic, and news events, there is a need for the use of broader data sets covering more aspects of food security, including different stakeholders. Future use of NLP in food security and public health requires the context of and interpretation by public health experts, with the potential to track dimensions or events related to food security to inform evidence-based decision-making in the public health area of food security.

## Appendix

Multimedia Appendix 1 Glossary of terms.

Multimedia Appendix 2 Additional figures including additional topic model distribution and proportion of topics by quarter.

Multimedia Appendix 3 Additional tables including sentiment of tweets across study period, topic analysis description, topic analysis by quarter and the sentiment of topics.

## Abbreviations

API: application programming interface
LDA: latent Dirichlet allocation
NLP: natural language processing
SNAP: Supplemental Nutrition Assistance Program
VADER: Valence Aware Dictionary and Sentiment Reasoner

## Data Availability

The data sets generated during and analyzed during this study are available from the corresponding author on reasonable request. To comply with ethical requirements, the data are not publicly available to ensure anonymity.
