# Supplementary material for: Using Natural Language Processing to Explore Social Media Opinions on Food Security: Sentiment Analysis and Topic Modeling Study
Source: J Med Internet Res. 2024 Mar 21;26:e47826. doi: 10.2196/47826 (PMC10995791; doi:10.2196/47826)
Supplement: Multimedia Appendix 1 [file jmir_v26i1e47826_app1.docx]

## Multimedia Appendix 1: Glossary of Terms

**Application programming interface (API):** An intermediary connection between software or computer applications that allows the software applications to communicate with each other and exchange data [8].

**Data science:** A field of science that uses a combination of mathematics and statistics, advanced analytics, artificial intelligence, specialised programming, and machine learning alongside subject matter expertise to uncover insights from data [9].

**Infodemic:** An unnecessary amount of information on a specific problem that is unfiltered that makes the ability to solve the problem more difficult [5].

**JavaScript Object Notation (JSON):** A lightweight data-interchange format that is used to send data between computers [10].

**Latent Dirichlet Allocation (LDA):** A generative probabilistic model that uses statistics to group words in a dataset into topics, where each topic in the dataset is characterised by a certain mixture of related words. LDA is a method used in topic modelling [7].

**Lexicon:** A type of dictionary used in natural language processing that contains information (semantic, grammatical, sentiment polarity) about individual words or word strings [11].

**Machine learning:** A data analysis method that is a branch of artificial intelligence where machines learn from and identify patterns in data and then make analytical model building decisions based on these learnings [12].

**Natural language processing (NLP):** Computational techniques used to learn, understand and produce human language content. NLP can use machine learning techniques to perform a range of textual analyses, such as tracking trending topics, identifying opinions and beliefs around different topics, and identifying different social networks of people [4].

**Sentiment analysis:** Sentiment analysis (or opinion mining) uses written natural language to analyse the opinions, sentiments, attitudes, and emotions embodied within text. A machine learning technique where typically either a subset of the text data is coded to assign sentiment, or a lexicon with words assigned to their corresponding sentiment, is used to build, and train a machine learning model to classify the sentiment of the text [6].

**Social media:** Web-based services that allow individuals, communities, and organisations to connect, interact and build a community by enabling them to create, co-create, modify, share and engage with user-generated content that is easily accessible [13].

**Stemming:** In natural language processing, the process of reducing words to their common base form (ie eating would be stemmed to ‘eat’) [14].

**Topic modelling:** A natural language processing technique that uses a probabilistic statistical model to create topics based on related words within a dataset [7].

**Twitter verification:** This definition is changing; however, at the time of data collection, verification referred to a system used by social networking platform Twitter to communicate the authenticity of a Twitter user’s account, particularly for notable figures and organisations, to distinguish these accounts from imposter accounts [15].
