# Supplementary material for: Using Natural Language Processing to Explore Social Media Opinions on Food Security: Sentiment Analysis and Topic Modeling Study
Source: J Med Internet Res. 2024 Mar 21;26:e47826. doi: 10.2196/47826 (PMC10995791; doi:10.2196/47826)
Supplement: Multimedia Appendix 2 [file jmir_v26i1e47826_app2.docx]

## Multimedia Appendix 2: Additional figures


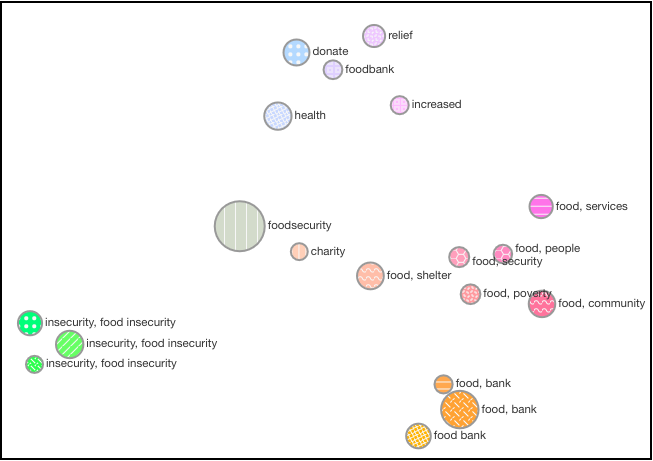


**Figure S1.** Topic model distribution for 19 topics as visualised on the topic map from BigML with 19 topics. Topic names are those automatically assigned by BigML. The size of the circle represents the probability of that topic within the dataset and the position of circles represents the thematic closeness of topics.


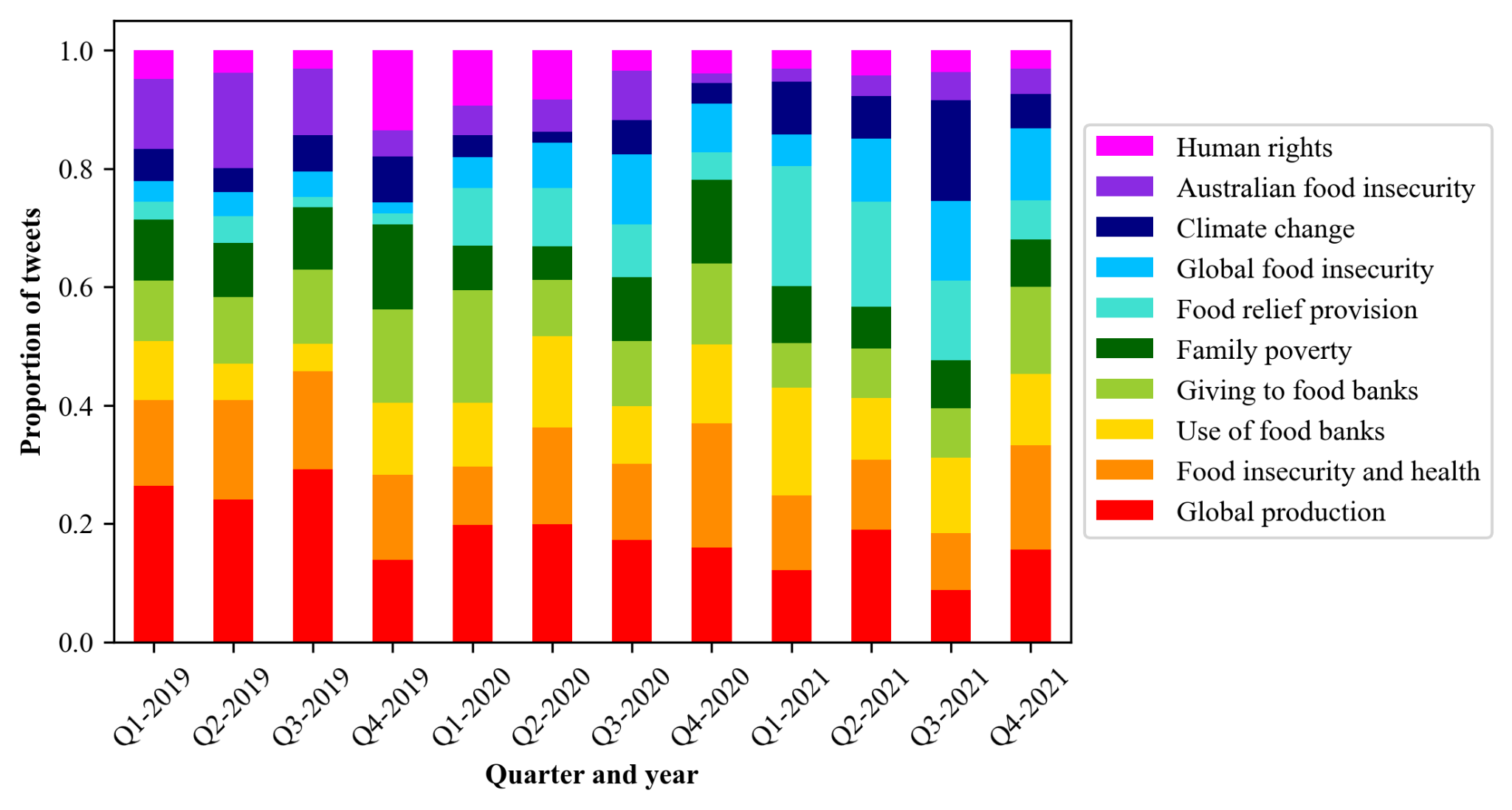


**Figure S2:** Proportion of highest probability topics by quarter and year.
