# Supplementary material for: Using Natural Language Processing to Explore Social Media Opinions on Food Security: Sentiment Analysis and Topic Modeling Study
Source: J Med Internet Res. 2024 Mar 21;26:e47826. doi: 10.2196/47826 (PMC10995791; doi:10.2196/47826)
Supplement: Multimedia Appendix 3 [file jmir_v26i1e47826_app3.docx]

## Multimedia Appendix 3: Additional Tables

Table S1. Sentiment of tweets across the study period years

| Sentiment | Total | 2019 | 2020 | 2021 |
| --- | --- | --- | --- | --- |
|  |  |  |  |  |
| Very positive | 1481 (3.9%) | 342 (3.2%) | 775 (5.1%) | 364 (3.0%) |
| Positive | 14,966 (39.3%) | 4115 (39.0%) | 5889 (38.5%) | 4962 (40.7%) |
| Neutral | 9060 (23.8%) | 2281 (21.6%) | 3636 (23.8%) | 3143 (25.7%) |
| Negative | 11,638 (30.6%) | 3610 (34.2%) | 4644 (30.3%) | 3384 (27.7%) |
| Very negative | 925 (2.4%) | 214 (2.0%) | 358 (2.3%) | 353 (2.9%) |

* Chi-square test of independence significant at p<.001 for differences between years

Table S2. Topic modeling description and comparison across the years (2019 to 2021; N=38,070).^a^

| Topic | Description | Key food security dimensions | Top 10 terms within the topic | Topic probability (%) | Total, n (%) | Number of tweets in 2019 (n=10,562), n (%) | Number of tweets in 2020 (n=15,302), n (%) | Number of tweets in 2021 (n=12,206), n (%) |
| --- | --- | --- | --- | --- | --- | --- | --- | --- |
| Global production | Production of food at a global level, including farming, agriculture, and environmental issues | Availability, stability, and sustainability | “Food security,” “agriculture,” “nutrition,” “sustainability,” “funding,” “read,” “farmers,” “production,” “global,” and “climate change” | 1.31 | 6656 (17.5) | 2246 (21.3) | 2810 (18.4) | 1600 (13.1) |
| Food insecurity and health | Food insecurity and health-related issues such as adverse health outcomes and the effect of the COVID-19 pandemic at a national Australian level | Access and use | “Food insecurity,” “insecurity,” “food,” “report,” “covid,” “health,” “hunger,” “national,” “Australia,” and “issue” | 1.19 | 5487 (14.4) | 1619 (15.3) | 2328 (15.2) | 1540 (12.6) |
| Use of food banks | Individual and household use of food banks | Access and stability | “Food,” “bank,” “food bank,” “line,” “week,” “support,” “students,” “hunger,” “pandemic,” and “remote” | 1.19 | 4534 (11.9) | 962 (9.1) | 1931 (12.6) | 1641 (13.4) |
| Giving to food banks | Supporting food banks and related charities through donation and volunteering | Availability, access, and agency | “Donate,” “support,” “help,” “local,” “foodbank,” “thanks,” “volunteers,” “please,” “local food,” and “raise” | 1.11 | 4561 (12) | 1391 (13.2) | 1999 (13.1) | 1171 (9.6) |
| Family poverty | Households experiencing poverty | Access and stability | “Families,” “poverty,” “live,” “children,” “day,” “food bank,” “money,” “bank,” “country,” and “feed” | 1.05 | 3670 (9.6) | 1251 (11.8) | 1415 (9.2) | 1004 (8.2) |
| Food relief provision | Government support for food relief at a national Australian level | Availability | “Relief,” “food relief,” “foodbank,” “community,” “help,” “increased,” “provide,” “demand,” “government,” and “services” | 0.93 | 3318 (8.7) | 274 (2.6) | 1282 (8.4) | 1762 (14.4) |
| Global food insecurity | Prevalence of food insecurity at a global level | Access and stability | “Food,” “people,” “million,” “food insecurity,” “insecurity,” “time,” “life,” “million people,” “levels,” and “world” | 0.88 | 2859 (7.5) | 321 (3) | 1239 (8.1) | 1299 (10.6) |
| Climate change | The effects of climate change on food security at a global level | Sustainability, availability, and stability | “Security,” “food security,” “climate,” “change,” “look,” “do not,” “climate change,” “month,” “people,” and “hungry” | 0.82 | 2506 (6.6) | 668 (6.3) | 540 (3.5) | 1298 (10.6) |
| Australian food insecurity | Food insecurity and political action within Australia at a national Australian level | Access and stability | “Food,” “Australians,” “food insecurity,” “insecurity,” “auspol,” “crisis,” “suffer,” “call,” “homeless,” and “access” | 0.79 | 2223 (5.8) | 991 (9.4) | 772 (5) | 460 (3.8) |
| Human rights | Food, water, and shelter as basic human rights at a global level | Access and stability | “Food,” “shelter,” “food shelter,” “charity,” “insecurity,” “care,” “research,” “system,” “water,” and “Govt” | 0.73 | 2256 (5.9) | 839 (7.9) | 986 (6.4) | 431 (3.5) |

^a^Chi-square test of independence significant at *P*<.001 for differences between the years; the topic model distribution is presented in Figure 2.

Table S3. Highest probability topic by quarter

| Topic | Quarter 1 2019 | Quarter 2 2019 | Quarter 3 2019 | Quarter 4 2019 | Quarter 1 2020 | Quarter 2 2020 | Quarter 3 2020 | Quarter 4 2020 | Quarter 1 2021 | Quarter 2 2021 | Quarter 3 2021 | Quarter 4 2021 |
| --- | --- | --- | --- | --- | --- | --- | --- | --- | --- | --- | --- | --- |
|  |  |  |  |  |  |  |  |  |  |  |  |  |
| Global production | 554 (26.4%) | 466 (24.1%) | 614 (29.1%) | 612 (13.8%) | 709 (19.8%) | 943 (19.9%) | 561 (17.3%) | 597 (16.0%) | 366 (12.2%) | 449 (19.0%) | 360 (8.8%) | 425 (15.6%) |
| Food insecurity and health | 306 (14.6%) | 325 (16.8%) | 351 (16.7%) | 637 (14.4%) | 352 (9.8%) | 774 (16.3%) | 415 (12.8%) | 787 (21.0%) | 379 (12.6%) | 280 (11.8%) | 399 (9.7%) | 482 (17.7%) |
| Use of food banks | 209 (10.0%) | 118 (6.1%) | 98 (4.7%) | 537 (12.1%) | 386 (10.8%) | 730 (15.4%) | 317 (9.8%) | 498 (13.3%) | 545 (18.1%) | 246 (10.4%) | 523 (12.7%) | 327 (12.0%) |
| Giving to food banks | 213 (10.1%) | 217 (11.2%) | 263 (12.5%) | 698 (15.8%) | 681 (19.0%) | 448 (9.5%) | 359 (11.1%) | 511 (13.7%) | 227 (7.6%) | 198 (8.4%) | 345 (8.4%) | 401 (14.7%) |
| Family poverty | 217 (10.3%) | 177 (9.2%) | 222 (10.5%) | 635 (14.4%) | 268 (7.5%) | 269 (5.7%) | 349 (10.8%) | 529 (14.1%) | 290 (9.7%) | 166 (7.0%) | 330 (8.0%) | 218 (8.0%) |
| Food relief provision | 64 (3.0%) | 88 (4.6%) | 37 (1.8%) | 85 (1.9%) | 350 (9.8%) | 471 (9.9%) | 289 (8.9%) | 172 (4.6%) | 608 (20.2%) | 420 (17.8%) | 553 (13.4%) | 181 (6.6%) |
| Global food insecurity | 71 (3.4%) | 78 (4.0%) | 90 (4.3%) | 82 (1.9%) | 188 (5.3%) | 362 (7.6%) | 382 (11.8%) | 307 (8.2%) | 159 (5.3%) | 253 (10.7%) | 556 (13.5%) | 331 (12.2%) |
| Climate change | 116 (5.5%) | 78 (4.0%) | 130 (6.2%) | 344 (7.8%) | 130 (3.6%) | 88 (1.9%) | 191 (5.9%) | 131 (3.5%) | 269 (9.0%) | 169 (7.1%) | 702 (17.1%) | 158 (5.8%) |
| Australian food insecurity | 249 (11.9%) | 312 (16.1%) | 236 (11.2%) | 194 (4.4%) | 180 (5.0%) | 258 (5.4%) | 271 (8.4%) | 63 (1.7%) | 67 (2.2%) | 84 (3.6%) | 192 (4.7%) | 117 (4.3%) |
| Human rights | 101 (4.8%) | 74 (3.8%) | 66 (3.1%) | 598 (13.5%) | 336 (9.4%) | 393 (8.3%) | 111 (3.4%) | 146 (3.9%) | 93 (3.1%) | 100 (4.2%) | 154 (3.7%) | 84 (3.1%) |

*Chi-square test of independence significant at p<.001 for differences between quarter

Table S4. Sentiment and top topic of tweets

| Topic | Very negative | Negative | Neutral | Positive | Very positive |
| --- | --- | --- | --- | --- | --- |
|  |  |  |  |  |  |
| Global production | 47 (0.7%) | 1291 (19.4%) | 1731 (26.0%) | 3262 (49.0%) | 325 (4.9%) |
| Food insecurity and health | 217 (4.0%) | 2885 (52.6%) | 1204 (21.9%) | 1138 (20.7%) | 43 (0.8%) |
| Use of food banks | 26 (0.6%) | 1029 (22.7%) | 1692 (37.3%) | 1727 (38.1%) | 60 (1.3%) |
| Giving to food banks | 36 (0.8%) | 660 (14.5%) | 872 (19.1%) | 2526 (55.4%) | 467 (10.2%) |
| Family poverty | 139 (3.8%) | 1530 (41.7%) | 783 (21.3%) | 1115 (30.4%) | 103 (2.8%) |
| Food relief provision | 6 (0.2%) | 237 (7.1%) | 936 (28.2%) | 1944 (58.6%) | 195 (5.9%) |
| Global food insecurity | 270 (9.4%) | 1620 (56.7%) | 431 (15.1%) | 520 (18.2%) | 18 (0.6%) |
| Climate change | 27 (1.1%) | 607 (24.2%) | 780 (31.1%) | 1067 (42.6%) | 25 (1.0%) |
| Australian food insecurity | 82 (3.7%) | 1052 (47.3%) | 331 (14.9%) | 600 (27.0%) | 158 (7.1%) |
| Human rights | 75 (3.3%) | 727 (32.2%) | 300 (13.3%) | 1067 (47.3%) | 87 (3.9%) |

*Chi-square test of independence significant at p<.001 for differences across sentiment
